# Efficacy and safety of direct oral anticoagulants in patients with atrial fibrillation combined with chronic kidney disease: a systematic review and meta-analysis

**DOI:** 10.1186/s12959-024-00608-5

**Published:** 2024-04-29

**Authors:** Yaodi Li, Shuyi Wu, Jintuo Zhou, Jinhua Zhang

**Affiliations:** Department of Pharmacy, Fujian Maternity and Child Health Hospital College of Clinical Medicine for Obstetrics & Gynecology and Pediatrics, Fujian Medical University, #18 Daoshan Road, Fuzhou, 350001 China

**Keywords:** Direct oral anticoagulants, Chronic kidney disease, Kidney failure, Atrial fibrillation, meta-analysis

## Abstract

**Background:**

Currently published studies have not observed consistent results on the efficacy and safety of direct oral anticoagulants (DOACs) use in patients with chronic kidney disease (CKD) combined with atrial fibrillation (AF). Therefore, this study conducted a meta-analysis of the efficacy and safety of DOACs for patients with AF complicated with CKD.

**Methods:**

Database literature was searched up to May 30, 2023, to include randomized controlled trials (RCT) involving patients with AF complicated with CKD DOACs and vitamin K antagonists (VKAs). Stroke, systemic embolism (SE), and all-cause mortality were used as effectiveness indicators, and major bleeding, intracranial hemorrhage (ICH), fatal bleeding, gastrointestinal bleeding (GIB), and clinically relevant non-major bleeding (CRNMB) were used as safety outcomes.

**Results:**

Nine RCT studies were included for analysis according to the inclusion criteria. Results of the efficacy analysis showed that compared with VKAs, DOACs reduced the incidence of stroke/SE (OR = 0.75, 95% CI 0.67–0.84) and all-cause deaths (OR = 0.84, 95% CI 0.75–0.93) in patients with AF who had comorbid CKD. Safety analyses showed that compared with VKAs, DOACs improved safety by reducing the risk of major bleeding (OR = 0.76, 95%CI 0.65–0.90), ICH (OR = 0.46, 95%CI 0.38–0.56), and fatal bleeding (OR = 0.75, 95%CI 0.65–0.87), but did not reduce the incidence of GIB and CRNMB.

**Conclusion:**

Compared with VKAs, DOACs may increase efficacy and improve safety in AF patients with CKD (90 ml/min> Crcl≥15 ml/min), and shows at least similar efficacy and safety in AF patients with Kidney failure (Crcl<15 ml/min).

**Supplementary Information:**

The online version contains supplementary material available at 10.1186/s12959-024-00608-5.

## Introduction

Published studies have yielded inconsistent findings regarding the effectiveness and safety of DOACs in patients with AF and CKD. Patients with atrial fibrillation (AF) are susceptible to stroke or thromboembolic events due to increased heart rate, enlarged atria, and the special structure of the left atrium, which can lead to stagnation of blood flow [1]. To prevent thromboembolism, oral anticoagulants (OACs), including VKAs and direct oral anticoagulants (DOACs), are one of the preferred treatments for patients at risk of thromboembolism [2]. Compared with warfarin, DOACs is gradually being widely used in the clinic because of its fixed-dose, shorter half-life, and rapid elimination after discontinuation [3]. In addition, it has been shown that compared with warfarin, DOACs used in patients with atrial fibrillation with normal renal function can lead to a significant reduction in the risk of thrombosis and hemorrhage [4, 5], and the efficacy and safety have been clinically proven to be superior to that of warfarin [6, 7].

VKAs are metabolized by the liver and no dose adjustment is required in renal insufficiency. In contrast, DOACs has varying degrees of renal clearance, with approximately 80% of dabigatran, 50% of edoxaban, 35% of rivaroxaban, and 27% of apixaban excreted [8]. Owing to the exclusion of patients with advanced-stage CKD from phase 3 clinical trials of DOACs, the utilization of DOACs has lagged behind in this specific population. In dialysis patients, the utilization of VKAs poses heightened risks such as renal calcification and diminished platelet production due to renal failure, thus increasing the likelihood of bleeding within the typical INR range compared to the general population. Patients undergoing hemodialysis are subject to systemic heparin anticoagulant therapy during the treatment period. However, studies assessing bleeding complications in hemodialysis patients treated with AVKs or DOACs have not taken into consideration the extent of heparinization during hemodialysis treatment. Therefore, the efficacy and safety of DOACs in AF patients combined with CKD, particularly in cases involving kidney failurehas been controversial. The current literature review indicates that, in patients with moderate CKD, dabigatran and apixaban exhibit superior efficacy over VKAs in reducing the incidence of stroke and systemic embolism. However, no statistically significant differences emerge between apixaban, rivaroxaban, and VKAs in this regard. In terms of major bleeding risk reduction, edoxaban, apixaban, and VKAs demonstrate more favorable outcomes compared to VKAs alone. Conversely, no notable distinction is observed between rivaroxaban and dabigatran etexilate in this aspect [9]. A meta-analysis conducted by T. Ha et al. found insufficient evidence to determine the superiority of VKAs or DOACs in AF patients with advanced CKD [10]. Another study showed that DOACs were not associated with a reduced risk of thromboembolism in AF patients on long-term dialysis, whereas VKAs, dabigatran, and rivaroxaban were associated with a significantly higher risk of bleeding compared with apixaban and no anticoagulants [11]. However, one study showed that DOACs was significantly more effective and safer than VKAs in patients with CKD or ESRD combined with AF [12]. In addition, a meta-analysis of the use of DOACs and VKAs in end-stage dialysis patients showed at least similar efficacy and safety [13].

Therefore, to better investigate the efficacy and safety of DOACs use in patients with CKD combined with AF, the present study conducted a systematic review and meta-analysis of the available evidence from randomized controlled trials (RCTs) to inform clinical medication decisions.

## Methods

We conducted a systematic review and meta-analysis of DOACs and VKAs for patients with AF comorbid CKD. CKD is defined as abnormalities of kidney structure or function, present for > 3 months, with implications for health. CKD is classified based on Cause, GFR category (G1–G5), and Albuminuria category (A1–A3) [14]. Renal insufficiency was defined as patients with CrCl < 95 ml/min, and patients with CrCl < 15 ml/min were defined as patients with kidney failure [15]. This study was conducted under the Preferred Reporting Initiative (PRISMA), registration number: CRD42023451323 [16].

### Search strategies

The PubMed and Web of Science databases were searched and the time frame was from the creation of the database to May 31, 2023. To ensure a comprehensive literature search, we also identified additional studies by searching the reference lists of the literature. Search words: (“dabigatran” or “rivaroxaban” or “apixaban” or “edoxaban” or “NOAC” or “DOAC” or “non-vitamin K antagonist oral anticoagulantacting” or “novel oral anticoagulant” and “warfarin” or “coumadin” or “vitamin K antagonist”) and (“renal insufficient” or “kidney disease” or “chronic renal insufficiency” or “end-stage renal disease” or “renal dialysis or hemodialysis” and “atrial fibrillation” or “kidney failure”). Detailed search strategies for each database are provided in Table S1.

### Study selection

 Inclusion criteria: (1) RCT; (2) The study was conducted in AF patients with CKD; (3) DOACs, including comparative studies of apixaban, rivaroxaban, edoxaban, and dabigatran with VKAs. Both control and experimental groups reported at least one bleeding or thrombosis occurrence data; (4) Full text was available and relevant data could be extracted.

Exclusion criteria: (1) Patients with AF not comorbid with CKD; (2) Duplicate studies or incomplete experimental data.

### Data extraction and study outcomes

Data extraction was done independently by two researchers (YL and SW). If there was a dispute, it will be discussed and resolved by a third researcher (JZ) to reach a consensus. Regarding missing data, we endeavor to communicate with the authors of the primary studies in an effort to obtain any unavailable data. If this proves unfeasible, we resort to the exclusion of studies with missing data. It is crucial to note, however, that this exclusionary approach may introduce selection bias if the missing data not missing completely at random. Furthermore, to ensure the reliability and robustness of our findings, we undertake a sensitivity analysis, scrutinizing the impact of varying scenarios on our results.

The following data were extracted from each study: study information (authors, year of publication), study characteristics (study population, sample size, duration of follow-up), intervention, and outcome indicators. Efficacy indicators were stroke, SE, and all-cause deaths. Safety indicators were major bleeding, intracranial hemorrhage (ICH), fatal bleeding, gastrointestinal bleeding (GIB), clinically relevant nonmajor bleeding (CRNMB), and minor bleeding.

### Quality assessment

Using the Cochrane Collaboration Risk Assessment Tool [17], two authors independently evaluated each paper for bias in seven areas: generation of randomized sequences, allocation concealment, blinding of subjects and investigators, blinding of outcome evaluations, completeness of outcome data, selective reporting of outcomes, and other biases. The level of risk of bias was evaluated as “high”, “low”, and “unclear”. If there is a dispute, another researcher (JZ) will evaluate it and help to solve the problem.

### Statistical analysis

Data on the incidence of all-cause death, stroke, SE, major bleeding, ICH, fatal bleeding, GIB, CRNMB, and minor bleeding were extracted for the inclusion of the experimental group and the control group. Forest plots were made using Review Manager 5.3 software. *P*-value, odds ratio (OR), and 95% confidence interval (Cl) were used as indicators of statistical differences in the comparison of the two groups. *P*-value < 0.05 and 95% Cl not containing 1 were considered as statistically significant differences. Statistical heterogeneity of the included studies was assessed using the Cochrane q-test *p*-value and *I*² statistic, where a Cochrane q-test *p*-value < 0.1 or *I*² value > 50% indicated significant heterogeneity. Fixed-effected model was used to calculate the pooled ORs and its 95% confidence interval (CI) if q-test *p*-value > 0.10 and *I*^2^ < 50%. Otherwise, the random-effect model was applied.

Sensitivity analyses and subgroup analyses were also performed to look for sources of heterogeneity. In secondary analyses, data on dabigatran were excluded and the meta-analysis was re-run considering that the direct thrombin inhibitor dabigatran has the highest rate of renal excretion and renal function has a greater impact on it. In addition, to analyze whether DOACs and renal function levels affect the study indexes, subgroup analyses were performed according to the type of DOACs and the level of renal function of the patients. Since less than ten papers were included in this study, publication bias detection was not performed.

## Results

### Literature search

**Fig. 1:**
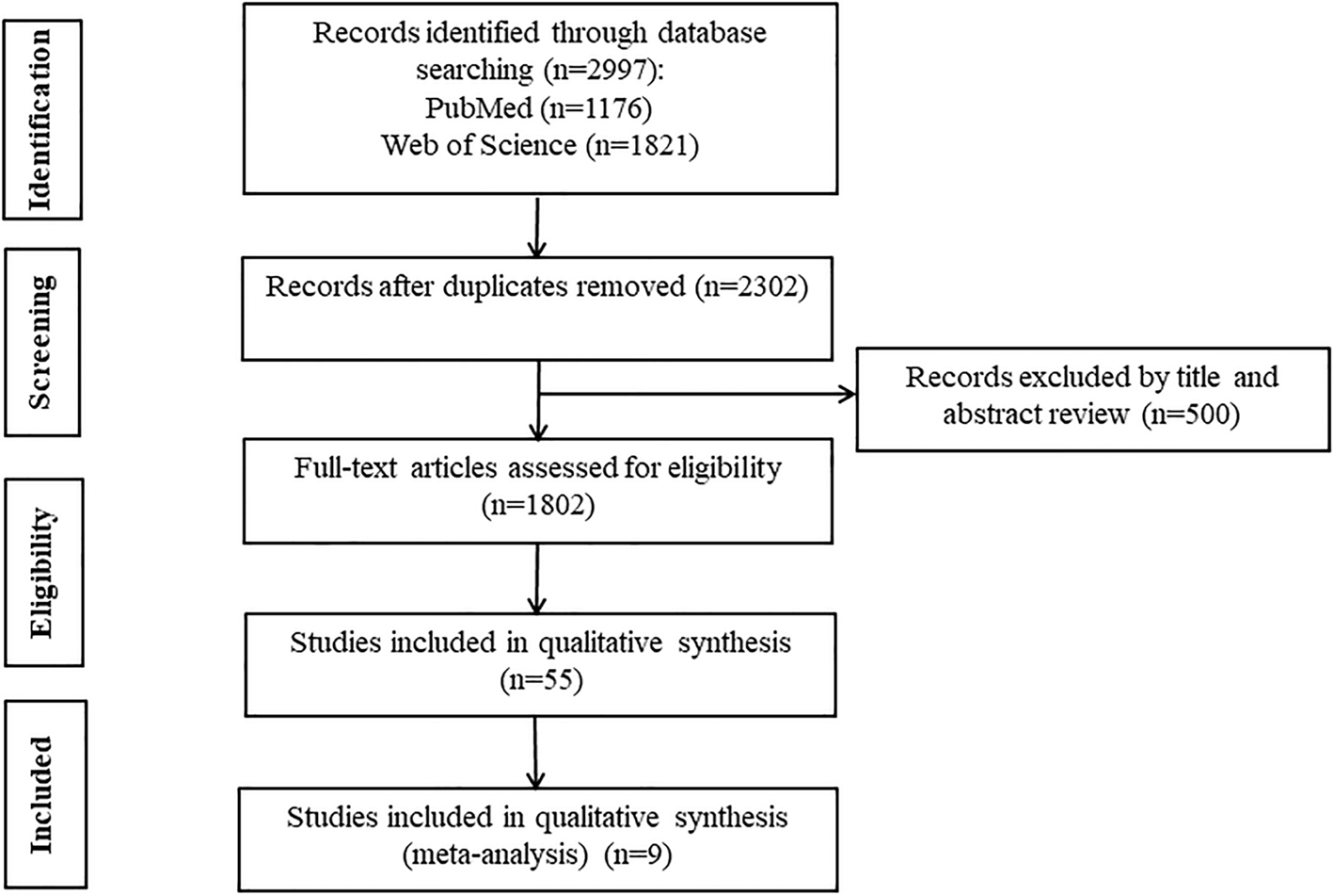
Flow chart for inclusion of studies

According to the search strategy, a total of 2997 papers were included, 695 duplicates were removed, and 500 papers of special types (review, case, letter, guideline, comment, animal) were removed. After reading the titles and abstracts and removing uncontrolled studies, reviews, and literature that could not be accessed in full text, the remaining 55 could be downloaded in full text for reading. After removing 25 of the cohort studies, as well as 21 of the literature with incomplete data that could not be extracted as relevant, and 1 of the literature with data that could not be transformed [18], the remaining 9 randomized controlled studies were included in the meta-analysis [19–27] The flow chart for inclusion in the study is shown in Fig. 1.

### Baseline characteristics of included studies

 The baseline characteristics of the included studies are shown in Table 1. Nine randomized controlled studies with 15 subgroups were included in this study. The experimental group drugs DOACs included apixaban [19, 25–27], rivaroxaban [20, 22, 24], dabigatran [21], and edoxaban [23]. The control drugs were dose-adjusted VKAs with INR values controlling between 2 and 3 except in literature where the control drug was warfarin (or matching placebo) [19] or phenprocoumon [27]. A total of 47,298 people were included, including 26,245 in the DOACs group and 21,053 in the VKAs group Table 2.

**Table 1:** Baseline characteristics of the included studies

**Study**	**Indication**	**Renal function (mL/min)**	**Group **	**DOACs**	**Control**	**Number (n)**		**Major bleeding/n**		**GIB/n**		**ICH/n**		**Fatal bleeding**		**CRNMB**		**Minor bleeding**		**Stroke**		**Stroke or SE**		**SE**		**All-caused death**		**Follow-up**
						**D**	**C**	**D**	**C**	**D**	**C**	**D**	**C**	**D**	**C**	**D**	**C**	**D**	**C**	**D**	**C**	**D**	**C**	**D**	**C**	**D**	**C**	
Hohnloser et al. 2012 [19]	patients with AF	GFR>50–80	^a1^	Apixaban 5 mg twice daily or Apixaban 2.5 mg twice daily	Warfarin (or matching placebo) was provided as 2 mg tablet	4334	3253	157	199													87	116			244	251	
		GFR≤50	^a2^	Apixaban 5 mg twice daily or Apixaban 2.5 mg twice		1641	1376	73	142													54	69			188	221	
Hori et al. 2012 [20]	AF Patients With Moderate Renal Impairment	(CrCl 30–49		rivaroxaban 10mg once daily	Warfarin (INR 2-3)	141	143	9	11	3	2	2	4															
Hijazi et al. 2013 [21]	Patients with AF	CrCl 50-80	^b1^	Dabigatran 110 mg bid	warfarin	2803	2989	158	209			14	49	74	107							94	103			175	244	> 2 years
			^b2^	Dabigatran 150 mg bid	(INR2.0-3.0)	2852		188				22		87								70				198		
		CrCl 30-49	^b3^	Dabigatran 110 mg bid		1196	1126	122	116			11	26	56	61							52	57			176	143	
			^b4^	Dabigatran 150 mg bid		1232		129				9		60								36				159		
B. Fordyce et al. 2016 [22]	Patients with AF	CrCl 30-49		Rivaroxaban 15mg qd or 20 mg qd	Warfarin (INR2.0-3.0)	6253	6359	258	262			45	59	18	27	643	665					143	164			144	194	median 707 days
A. Bohula et al. 2016 [23]	patients with AF	CrCl 30-50	^c1^	Edoxaban 30 mg daily	Warfarin (INR2.0-3.0)	1379	1361	100	132	51	43	17	36	9	19			120	150	77	82			5	9	251	300	median 2.8 years
		CrCl50-95	^c2^	Edoxaban 60 mg daily		4060	4148	267	309	155	109	37	88	18	34			382	434	156	201			9	12	400	435	
Vriese et al. 2021 [24]	Patients with AF	Hemodialysis	^d1^	Rivaroxaban 10mg	VKA	46	44	6	10	9	12			3	11			16	13	4	9			0	0	30	32	median 1.88 years
			^d2^	Rivaroxaban and Vitamin K2		42		4		13				6				16		2				0		27		
W. Stanifer et al. 2020 [25]	Patients With AF	CrCl25-30		Apixaban 5mg twice daily or 2.5mg twice daily	Warfarin	136	133	7	19			0	4									6	10			33	32	median 1.8 years
D. Pokorney et al. 2022 [26]	patients with AF	Hemodialysis		Apixaban5mg twice daily or 2.5mg twice daily	Warfarin	82	72	9	7	4	7	1	1	1	2	14	10			2	2			0	0	21	13	median 330 days
Reinecke et al. 2023 [27]	Patients With AF	Hemodialysis		apixaban (2.5 mg BID)	VKA (phenprocoumon)	48	49	5	6							10	9			0	1					9	12	median 429 days

**Table 2:** Summary of results of validity and safety analyses

	**All-caused death**	**Stroke**	**Systemic embolism**	**Stroke or systemic embolism**	**Major bleeding**	**ICH**	**Fatal bleeding**	**Gastrointestinal bleeding**	**Clinically relevant**	**Minor bleeding**
Main analysis: mixed DOACs										
No. of paper	7	4	3	4	9	6	5	4	3	2
No. of study groop	13	6	5	8	15	10	10	6	3	4
* P*-value	0.0007	0.008	0.25	＜0.01	0.001	＜0.00001	0.0001	0.01	0.83	0.03
0R	0.84	0.79	0.67	0.75	0.76	0.46	0.75	1.3	0.99	0.87
95% CIs	0.75-0.93	0.67-0.94	0.34-1.33	0.67-0.84	0.65-0.9	0.38-0.56	0.65-0.87	1.07-1.58	0.88-1.11	0.77-0.99
* P*	0.01	0.35	0.63	0.07	＜0.0001	0.25	0.44	0.35	0.8	0.39
*I*^2^ statistic	53%	10%	0%	46%	73%	20%	0%	10%	0%	0%
Secondary analysis: factor Xa inhibitors										
No. of paper	7	4	3	3	8	5	4	4	3	2
No. of study groop	10	6	5	4	11	6	6	6	3	4
* P*-value	＜0.0001	0.008	0.25	0.006	0.001	0.0001	0.0002	0.06	0.83	0.02
0R	0.79	0.79	0.67	0.68	0.67	0.54	0.53	1.26	0.99	0.86
95% CIs	0.71-0.88	0.67-0.94	0.34-1.33	0.52-0.90	0.52-0.85	0.38-0.74	0.38-0.74	0.99-1.60	0.88-1.11	0.76-0.98
* P*	0.18	0.35	0.64	0.07	＜0.00001	0.27	0.76	0.35	0.8	0.43
*I*^2^ statistic	28%	10%	0%	57%	76%	22%	0%	10%	0%	0%
CKD (Crcl＞15ml/min) and AF Subgroup analysis by DOAC										
1) Rivaroxaban										
No. of study groop	1			1	2	2	1	1	1	
0R	0.75			0.88	0.99	0.76	0.68	1.53	0.98	
95% CIs	0.6-0.93			0.7-1.11	0.84-1.18	0.52-1.11	0.37-1.25	0.25-9.91	0.88-1.10	
2) Apixaban										
No. of study groop	3			3	3	1				
0R	0.71			0.58	0.47	0.11				
95% CIs	0.62-0.81			0.47-0.73	0.35-064	0.01-1.98				
3) Edoxaban										
No. of study groop	2	2	2		2	2	2	2		2
OR	0.87	0.82	0.67		0.82	0.43	0.51	1.39		0.86
95% CIs	0.73-1.02	0.69-0.99	0.34-1.33		0.70-0.97	0.32-0.60	0.32-0.82	1.12-1.72		0.76-0.97
3) Dabigatran										
No. of study groop	4			4	4	4	4			
OR	0.93			0.79	0.92	0.38	0.82			
95% CIs	0.76-1.13			0.67-0.93	0.82-1.03	0.28-0.51	0.70-0.97			
Kidney failure and AF Subgroup analysis by DOAC										
1) Rivaroxaban										
No. of study groop	2	2			2		2	2		2
0R	0.69	0.29			0.44		0.35	0.9		1.36
95% CIs	0.36-1.31	0.11-0.78			0.19-1.0		0.15-0.83	0.46-1.76		0.73-2.57
2) Apixaban										
No. of study groop	2	2			2	1	1	1	2	
0R	1.15	0.67			1.01	0.88	0.43	0.48	1.23	
95% CIs	0.63-2.10	0.12-3.64			0.45-2.25	0.05-14.27	0.04-4.87	0.13-1.7	0.63-3.38	
Subgroup analysis by CKD stage										
CrCl50-95ml/min										
No. of study groop	4	1		3	4	3	3			
OR	0.82	0.78		0.72	0.79	0.41	0.76			
95% CIs	0.75-0.90	0.63-0.97		0.52-1.01	0.64-0.96	0.31-0.53	0.62-0.92			
CrCl30-49ml/min										
No. of study groop	2	1		3	3	3	4			
OR	0.77	0.92		0.78	0.87	0.5	0.8			
95% CIs	0.67-0.89	0.67-1.27		0.61-1.01	0.68-1.12	0.34-0.74	0.64-1.01			
CrCl15-29ml/min										
No. of study groop	1			1	1	1	0			
OR	1.01			0.57	0.33	0.11				
95% CIs	0.58-1.71			0.2-1.61	0.13-0.8	0.01-1.98				
CrCl＜15ml/min										
No. of study groop	4	4		0	4	1	3			
OR	0.91	0.36			0.67	0.88	0.19			
95% CIs	0.59-1.40	0.15-0.84			0.38-1.2	0.05-14.27	0.06-0.56			

### Quality assessment

**Fig. 2:**
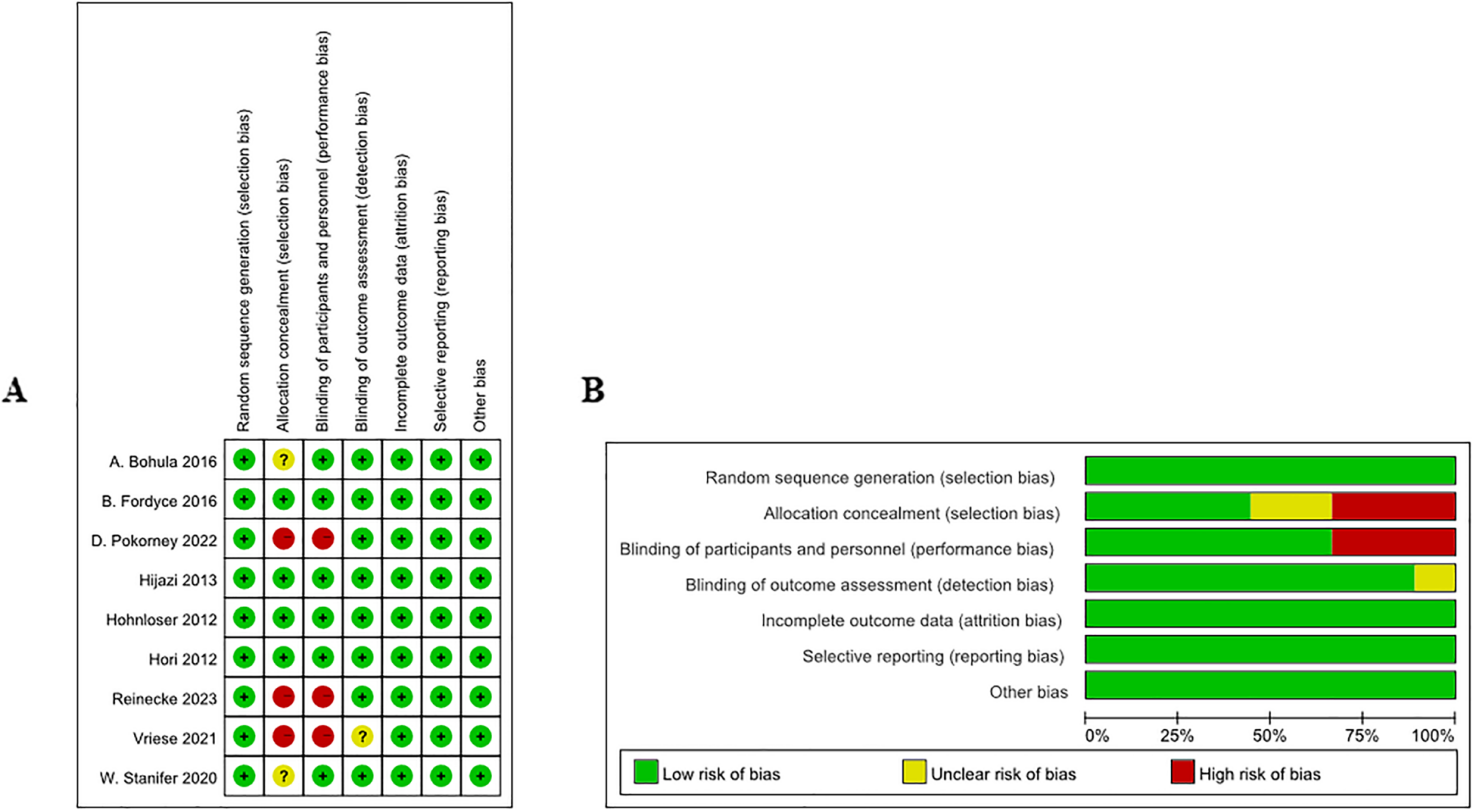
Risk of bias in each study. Green, low risk of bias; yellow, unclear risk of bias; and red, high risk of bias

Nine articles were at low risk for randomized sequence generation, completeness of outcome data, selective reporting of results, and other biases. Three articles were at high risk for allocation concealment and two were unclear. Three articles were at high risk for blinding of subjects and investigators, and one article was unclear about blinding of results (Fig. 2).

### Trial sequential analysis

**Fig. 3:**
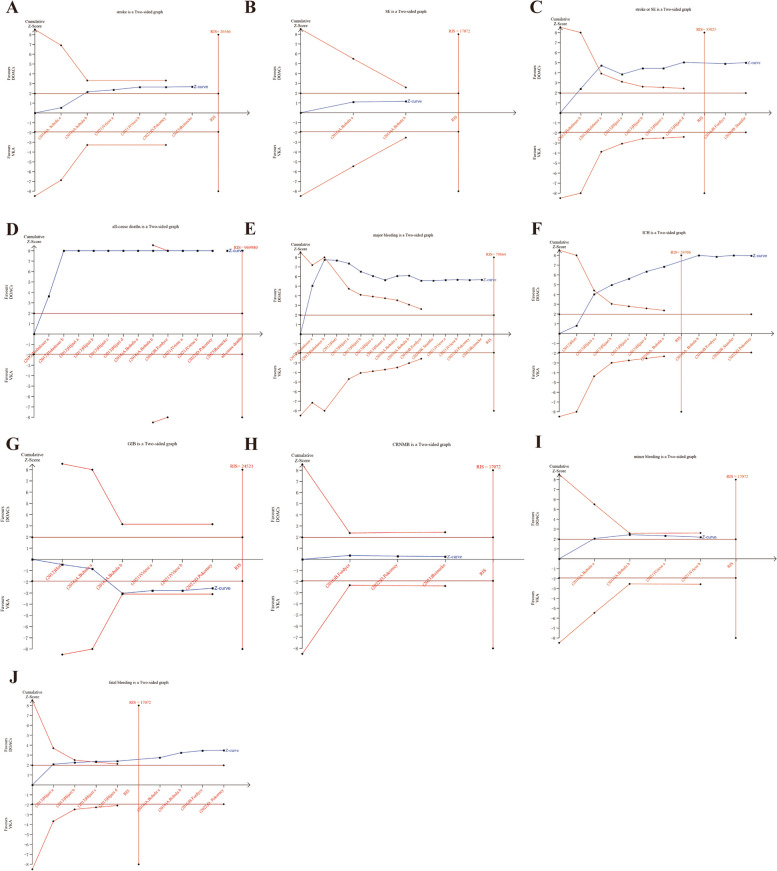
TSA results for various outcome measures in AF patients with CKD treated with DOACs versus VKAs. **A** stroke, **B** SE, **C** stroke or SE, **D** all-cause deaths, **E** major bleeding, **F** ICH, **G** GIB, **H** CRNMB, **I** minor bleeding, **J** fetal bleeding

Trial Sequential Analysis (TSA) was conducted in this study for various outcome indicators. A two-sided type I error probability (α) of 0.05 and type II error probability (β) of 0.20 were established, along with the definition of the Required Information Size (RIS). The control group’s incidence was computed using the data from the included studies. The TSA results showed the cumulative Z curves for stroke or SE, ICH and fetal bleeding crossed both the conventional and TSA boundaries and reached the the required information size (RIS), indicating that the meta-analysis results were stable and statistically significant (Fig. 3C, F, J). The cumulative Z curves for all-cause death and major bleeding crossed the traditional threshold and TSA threshold, further confirming the credibility of the synthesized data (Fig. 3D, E). The cumulative Z curves for stroke, minor bleeding, GIB crossed conventional test boundary; however, they did not cross Alpha-spending boundary, nor did it reach the required information size (Fig. 3A, I, G). And the cumulative Z curves for SE and CRNMB did not cross trial sequential monitoring boundaries, and the sample size did not reach the RIS, suggesting no conclusive evidence to support a statistically significant difference in reducing SE and CRNMB, and larger randomized controlled trials are warranted to further investigate these outcomes (Fig. 3B, H).

### Efficacy analysis

Comparison of efficacy metrics for stroke, SE, and all-cause death between DOACs and VKAs groups.

**Fig. 4:**
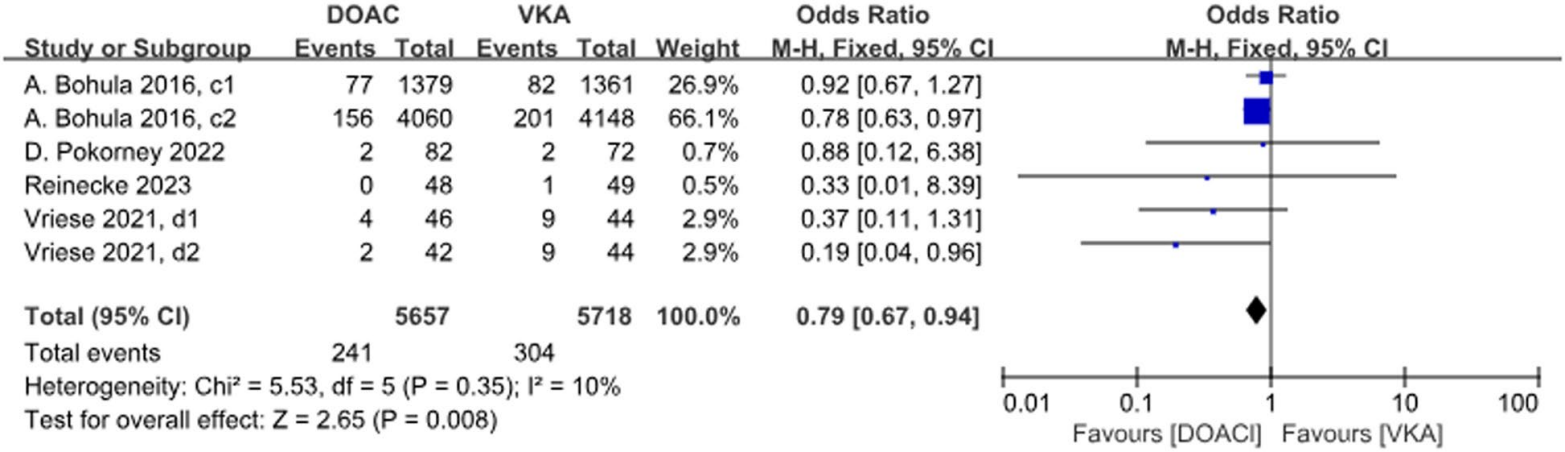
Forest plot for stroke in AF patients with CKD treated with DOACs versus VKAs. c1: CrCl 30-50 ml/min, edoxaban 30 mg daily; c2: CrCl 50-95 ml/min, edoxaban 60 mg daily; d1: CrCl＜15 ml/min, rivaroxaban 10mg daily; d2: CrCl＜15 ml/min, rivaroxaban and vitamin K2

4 studies with a total of 6 data sets compared stoke, resulting in a 21% reduction in incidence with DOACs, although there was no statistically significant difference (*P* = 0.008, OR = 0.79, 95% CI 0.67–0.94) and no significant heterogeneity between groups (*P* = 0.35, *I*^2^ = 10%) (Fig. 4).

**Fig. 5:**
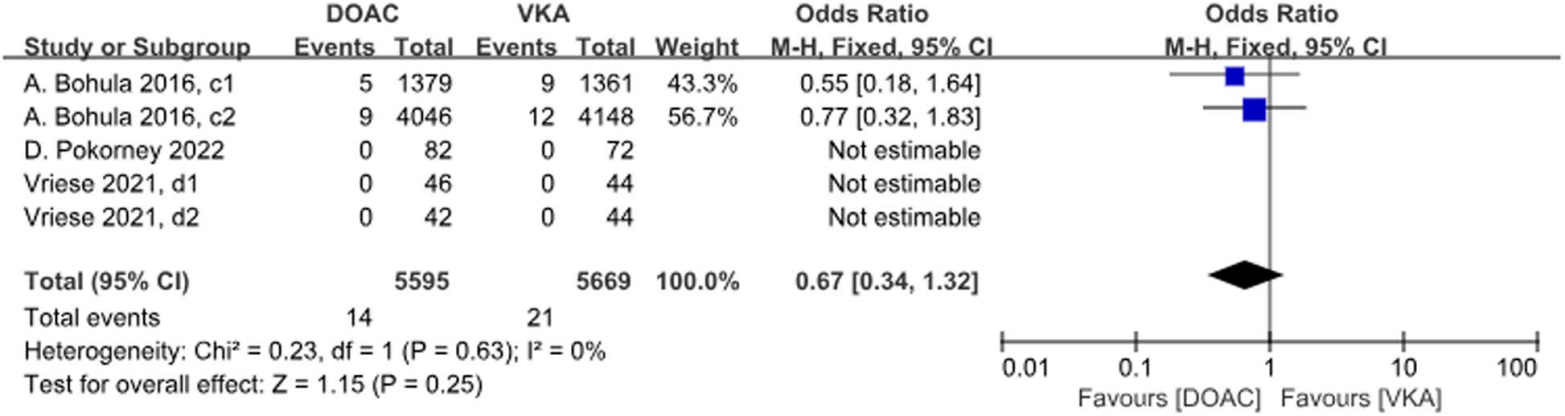
Forest plot for SE in AF patients with CKD treated with DOACs versus VKAs. c1: CrCl 30-50 ml/min, edoxaban 30 mg daily; c2: CrCl 50-95 ml/min, edoxaban 60 mg daily; d1: CrCl＜15 ml/min, rivaroxaban 10mg daily; d2: CrCl＜15ml/min, rivaroxaban and vitamin K2

 Three studies with a total of 5 data sets compared SE, resulting in a 33% reduction in the incidence of DOACs, although there was no statistically significant difference in the results (*P* = 0.25, OR = 0.67, 95%CI 0.34–1.32), and there was no significant heterogeneity between the groups (*P* = 0.63, *I*^2^ = 0%) (Fig. 5).

**Fig. 6:**
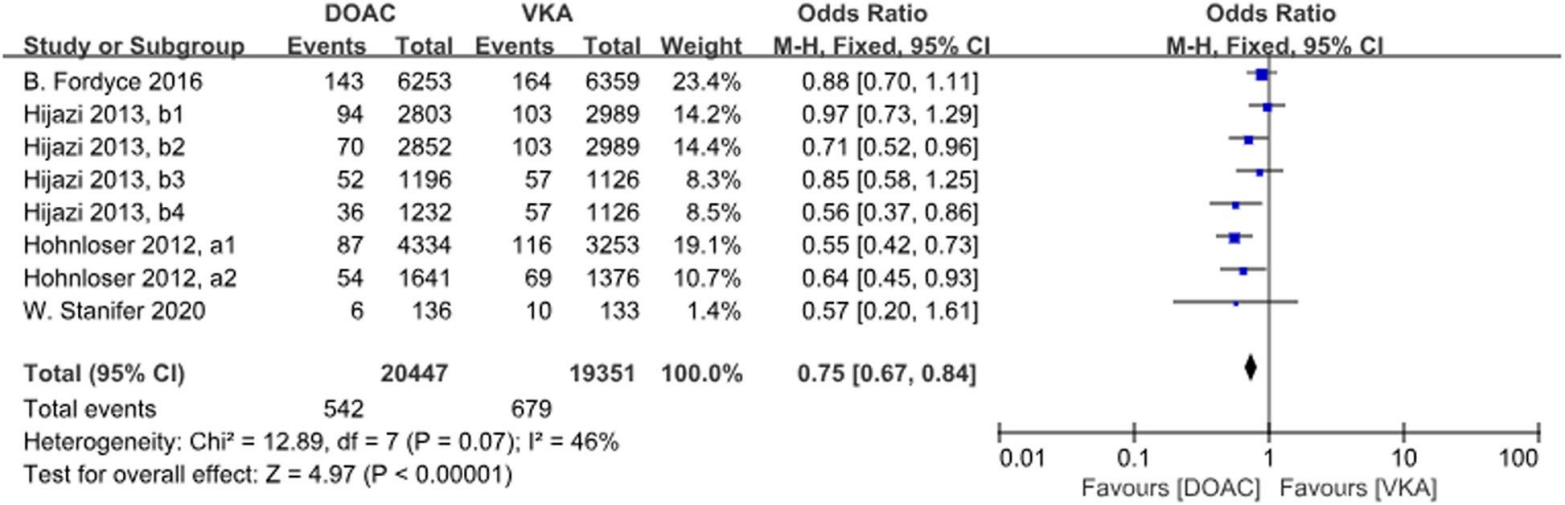
Forest plot for stroke or SE in AF patients with CKD treated with DOACs versus VKAs. a1: CrCl 51-80 ml/min, apixaban 5 mg twice daily or 2.5 mg twice daily; a2: CrCl≤50 ml/min, apixaban 5 mg twice daily or 2.5 mg twice daily; b1: CrCl 50-80 ml/min, dabigatran 110 mg twice daily; b2: CrCl 50-80 ml/min, dabigatran 150 mg twice daily; b3: CrCl 30-49 ml/min, dabigatran 110 mg twice daily; b4: CrCl 30-49 ml/min, dabigatran 150 mg twice daily

 There were 4 studies with a total of 8 data sets comparing stroke or SE. DOACs reduced the incidence by 25% compared with VKAs, a statistically different result (*P* < 0.001, OR = 0.75, 95% CI 0.67–0.84), with no heterogeneity between groups (*P* = 0.07, *I*^2^ = 46%) (Fig. 6).

**Fig.7:**
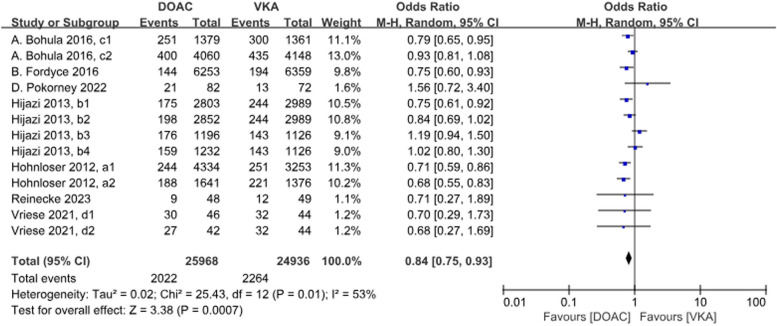
Forest plot for all-cause deaths in AF patients with CKD treated with DOACs versus VKAs. a1: CrCl 51–80 ml/min, apixaban 5 mg twice daily or 2.5 mg twice daily; a2: CrCl ≤ 50 ml/min, apixaban 5 mg twice daily or 2.5 mg twice daily; b1: CrCl 50–80 ml/min, dabigatran 110 mg twice daily; b2: CrCl 50–80 ml/min, dabigatran 150 mg twice daily; b3: CrCl 30–49 ml/min, dabigatran 110 mg twice daily; b4: CrCl 30–49 ml/min, dabigatran 150 mg twice daily; c1: CrCl 30–50 ml/min, edoxaban 30 mg daily; c2: CrCl 50–95 ml/min, edoxaban 60 mg daily; d1: CrCl<15 ml/min, rivaroxaban 10 mg daily; d2: CrCl<15 ml/min, rivaroxaban and vitamin K2

 A total of 13 data sets from 7 studies compared all-cause deaths. The results were statistically significant when comparing the DOACs group to the VKAs group, with a 16% reduction in incidence with DOACs (*P* = 0.0007, OR = 0.84, 95% CI 0.75–0.93), with heterogeneity between groups (*P* = 0.01, *I*^2^ = 53%) (Fig. 7).

### Safety analysis

Safety metrics such as major bleeding, ICH, and fatal bleeding were compared between the DOACs and VKAs groups.

**Fig. 8:**
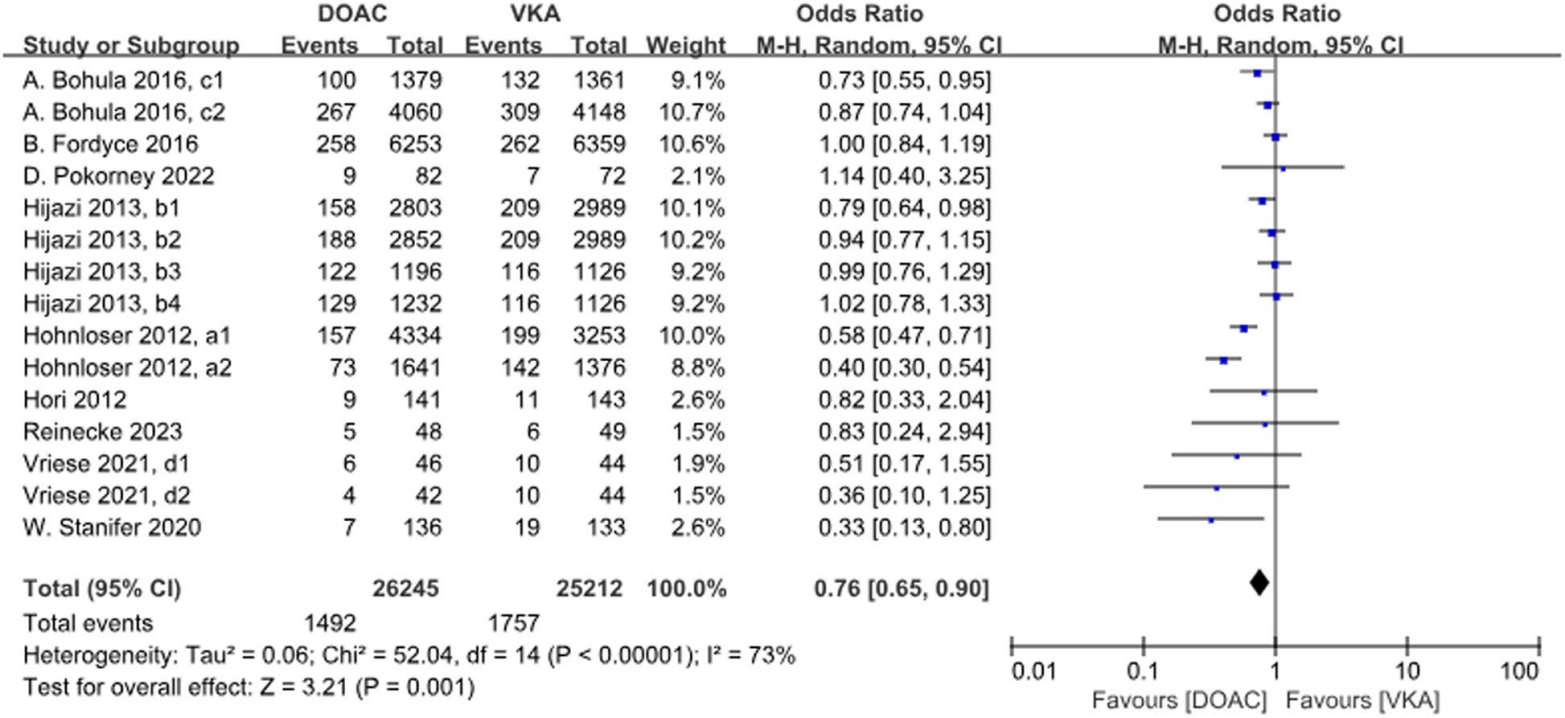
Forest plot for major bleeding in AF patients with CKD treated with DOACs versus VKAs. a1: CrCl 51–80 ml/min, apixaban 5 mg twice daily or 2.5 mg twice daily; a2: CrCl ≤ 50 ml/min, apixaban 5 mg twice daily or 2.5 mg twice daily; b1: CrCl 50–80 ml/min, dabigatran 110 mg twice daily. b2: CrCl 50–80 ml/min, dabigatran 150 mg twice daily. b3: CrCl 30–49 ml/min, dabigatran 110 mg twice daily; b4: CrCl 30–49 ml/min, dabigatran 150 mg twice daily; c1: CrCl 30–50 ml/min, edoxaban 30 mg daily; c2: CrCl 50–95 ml/min, edoxaban 60 mg daily; d1: CrCl<15 ml/min, rivaroxaban 10 mg daily; d2: CrCl<15 ml/min, rivaroxaban and Vitamin K2

A total of 9 studies with 15 data sets compared the incidence of major bleeding and the results were statistically different. DOACs compared with VKAs reduced the incidence by 24% (*P* = 0.001, OR = 0.76, 95%CI 0.65–0.90), and there was heterogeneity between the groups (*P* < 0.001, *I*^2^ = 73%) (Fig. 8).

**Fig.9:**
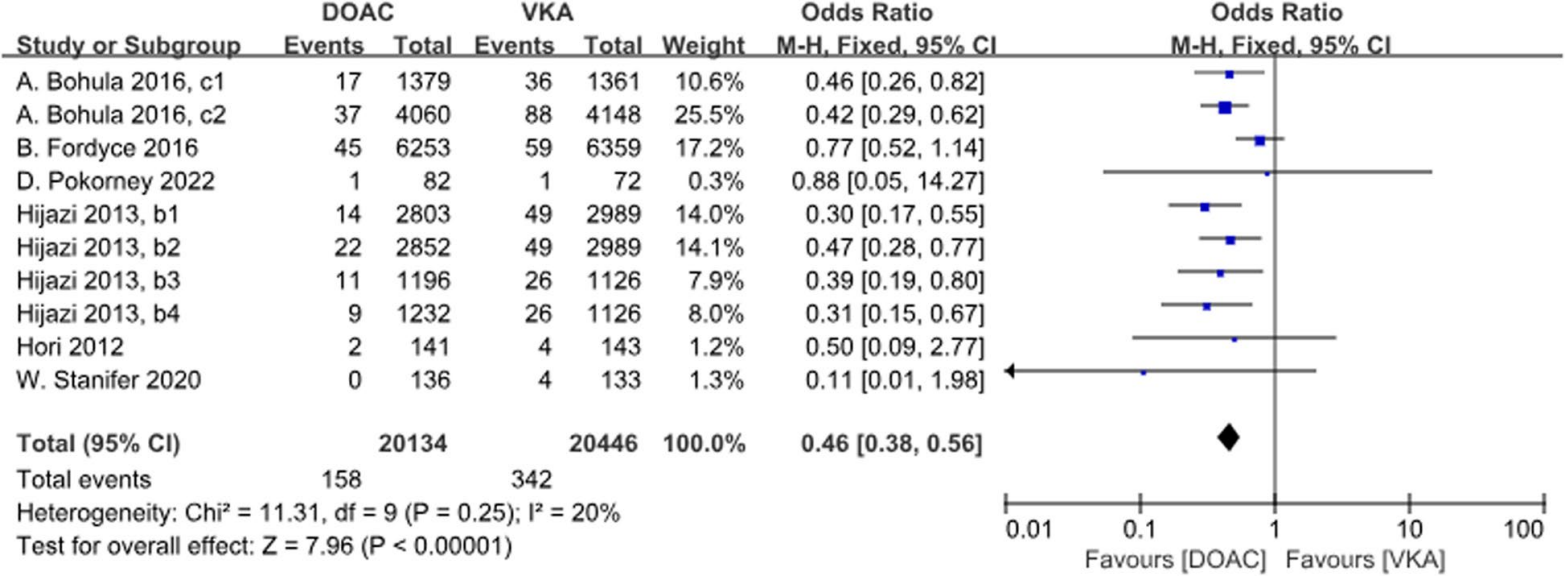
Forest plot for ICH in AF patients with CKD treated with DOACs versus VKAs. b1: CrCl 50–80 ml/min, dabigatran 110 mg twice daily. b2: CrCl 50–80 ml/min, dabigatran 150 mg twice daily. b3: CrCl 30–49 ml/min, dabigatran 110 mg twice daily; b4: CrCl 30–49 ml/min, dabigatran 150 mg twice daily; c1: CrCl 30–50 ml/min, edoxaban 30 mg daily; c2: CrCl 50–95 ml/min, edoxaban 60 mg daily

A total of 6 studies with 10 data sets compared ICH incidence. There was a significant difference between the two groups, with DOACs reducing the incidence by 54% (*P*<0.001, OR = 0.46, 95%CI 0.38–0.56) and no heterogeneity between groups (*P* = 0.25, *I*^2^ = 20%) (Fig. 9).

**Fig. 10:**
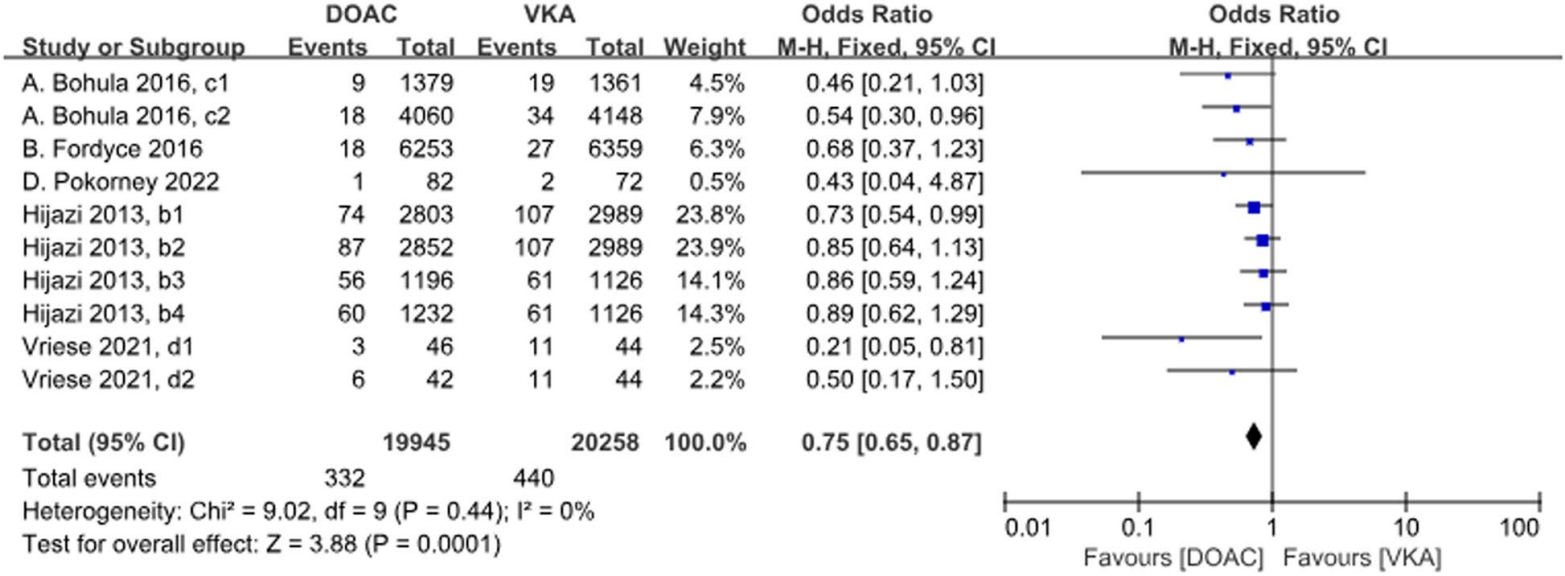
Forest plot for fetal bleeding in AF patients with CKD treated with DOACs versus VKAs. b1: CrCl 50–80 ml/min, dabigatran 110 mg twice daily. b2: CrCl 50–80 ml/min, dabigatran 150 mg twice daily. b3: CrCl 30–49 ml/min, dabigatran 110 mg twice daily; b4: CrCl 30–49 ml/min, dabigatran 150 mg twice daily; c1: CrCl 30–50 ml/min, edoxaban 30 mg daily; c2: CrCl 50–95 ml/min, edoxaban 60 mg daily; d1: CrCl<15 ml/min, rivaroxaban 10 mg daily; d2: CrCl<15 ml/min, rivaroxaban and Vitamin K2

A total of 5 studies with 10 datasets compared the incidence of fatal bleeding and there was a significant difference between the two groups, with DOACs reducing the incidence by 25% (*P* < 0.001, OR = 0.75, 95%CI 0.65–0.87). There was no heterogeneity between the groups (*P* = 0.44, *I*^2^ = 0%) (Fig. 10).

**Fig. 11:**
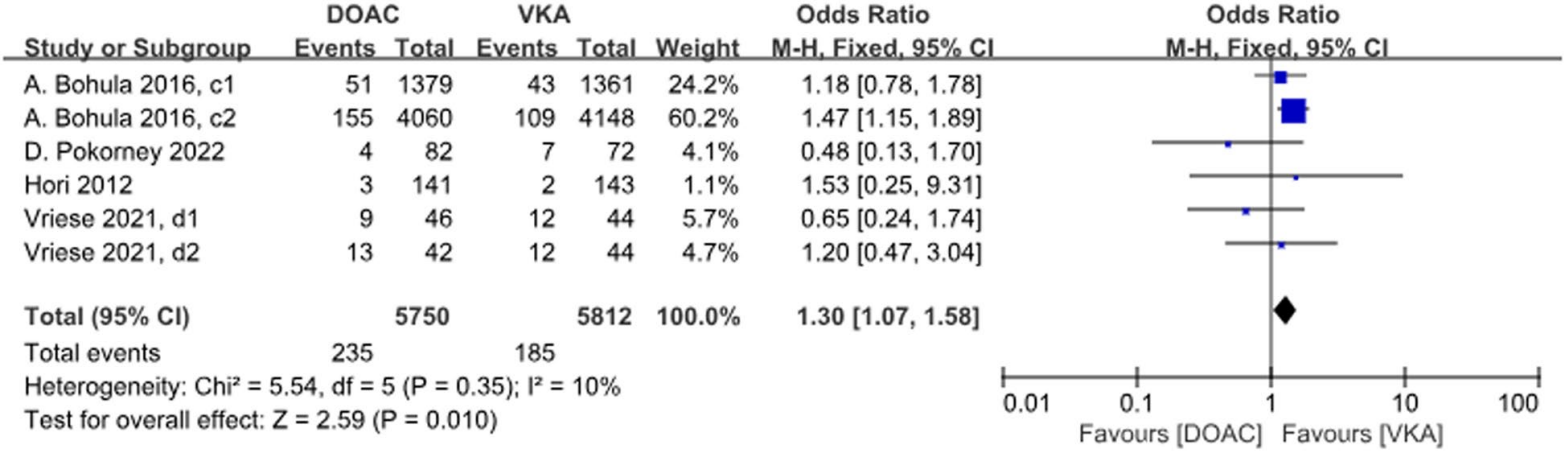
Forest plot for GIB AF patients with CKD treated with DOACs versus VKAs. c1: CrCl 30–50 ml/min, edoxaban 30 mg daily; c2: CrCl 50–95 ml/min, edoxaban 60 mg daily; d1: CrCl<15 ml/min, rivaroxaban 10 mg daily; d2: CrCl<15 ml/min, rivaroxaban and Vitamin K2

A total of 4 studies with 6 data sets compared GIB, with no significant difference between the two groups (*P* = 0.01, OR = 1.30, 95% CI 1.07–1.58) and no heterogeneity between the groups (*P* = 0.35, *I*^2^ = 10%) (Fig. 11).

**Fig. 12:**
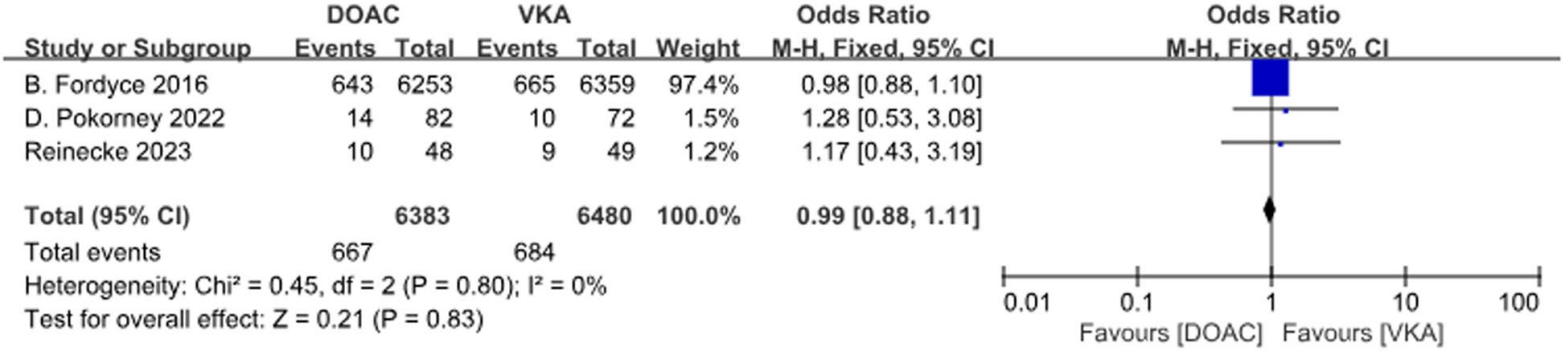
Forest plot for CRNMB in AF patients with CKD treated with DOACs versus VKAs

A total of 3 studies with 3 data sets compared CRNMB. There was no significant difference between the two groups (*P* = 0.83, OR = 0.99, 95% CI 0.88–1.11) and no heterogeneity between groups (*P* = 0.80, *I*^2^ = 0%). Forest plot results (Fig. 12).

**Fig. 13:**
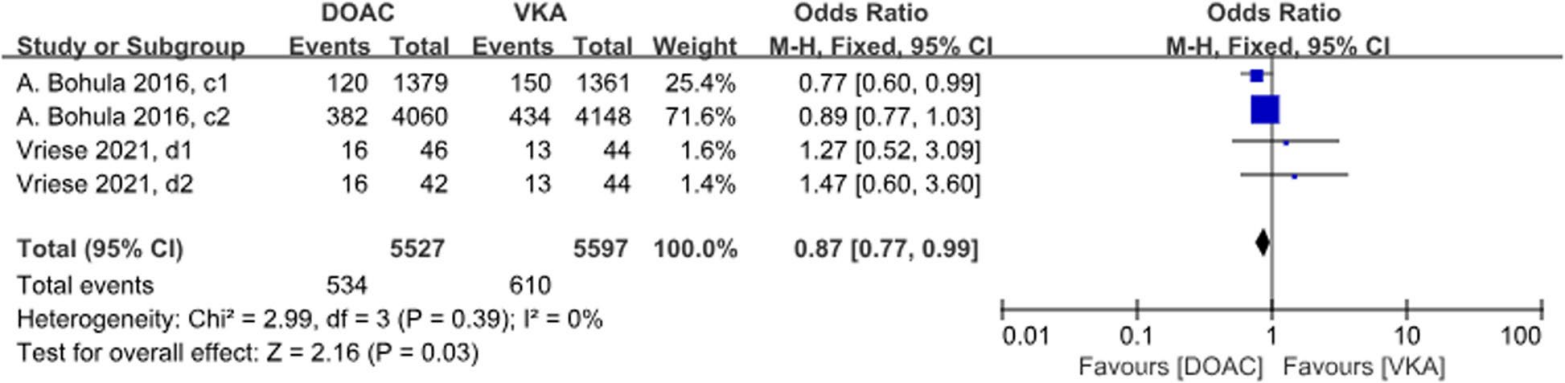
Forest plot for minor bleeding in AF patients with CKD treated with DOACs versus VKAs. c1: CrCl 30-50 ml/min, edoxaban 30 mg daily; c2: CrCl 50-95 ml/min, edoxaban 60 mg daily; d1: CrCl＜15 ml/min, rivaroxaban 10mg daily; d2: CrCl＜15 ml/min, rivaroxaban and Vitamin K2

A total of 2 studies with 4 sets of data compared minor bleeding. DOACs reduced the incidence by 13%, statistically different between the two groups (*P* = 0.03, OR = 0.87, 95% CI 0.77–0.99), with no heterogeneity between the groups (*P* = 0.39, *I*^2^ = 0%) (Fig. 13).

### Sensitivity analysis

There was heterogeneity in all-cause deaths. Further sensitivity analyses showed a decrease in *I*^2^ from 53 to 32% after the removal of the Hijazi et al. 2014c group [21], indicating that the CrCl 30–50 ml/min dabigatran 110 mg group was a possible source of heterogeneity in the all-cause deaths.

There was heterogeneity in the incidence of major bleeding. Further sensitivity analyses showed a decrease in *I*^2^ from 73 to 55% after removal in the Hohnloser et al. 2012, a1 group [19], from 73 to 68% after removal in the Hohnloser et al. 2012, a2 group [19], and from 73 to 19% after simultaneous removal in both groups, which suggests that there is a possible source of heterogeneity in major bleeding in the apixaban group.

### Subgroup analysis

For AF patients with CKD, subgroup analyses performed differently for DOACs showed that rivaroxaban and apixaban were superior in reducing all-cause deaths compared with VKAs, with rivaroxaban reducing the incidence by 25% (*P* = 0.01, OR = 0.75, 95% CI 0.60–0.93) and apixaban reducing the incidence by 29% (*P* < 0.001, OR = 0.71, 95% CI 0.62–0.81). There was no statistical difference between edoxaban and dabigatran (*P* = 0.09; *P* = 0.44). There was heterogeneity between groups (*P* = 0.1, *I*^2^ = 51.7%), suggesting that different DOACs may be a source of heterogeneity (Figure S1).

Subgroup analyses of AF patients with kidney failure differing for DOACs showed that neither rivaroxaban nor apixaban reduced the incidence of all-cause deaths compared with VKAs (Figure S2).

Subgroup analysis showed that DOACs reduced all-cause deaths in patients with CrCl 50–95 ml/min with heterogeneity between groups (*P*<0.001, OR = 0.82, 95% CI 0.75–0.90, *I*^2^ = 51%), and did not reduce all-cause deaths in patients with CrCl < 30 ml/min. There was no heterogeneity between groups (*P* = 0.69, *I*^2^ = 0%) (Figure S3).

For AF patients with CKD, subgroup analyses differing according to DOACs showed that apixaban significantly reduced the incidence of major bleeding compared with VKAs (*P* < 0.001, OR = 0.47, 95% CI 0.34–0.64); edoxaban reduced the incidence of major bleeding by 18% (*P* = 0.02, OR = 0.82, 95% CI 0.70–0.97), and no significant difference between rivaroxaban and dabigatran (*P* = 0.95; *P* = 0.14). Heterogeneity existed between the different DOACs study groups (*P* = 0.0004, *I*^2^ = 83.7%), suggesting that the different DOACs may be the source of the heterogeneity in major bleeding (Figure S4).

Subgroup analysis of AF patients with Kidney failure with different DOACs showed that neither rivaroxaban nor apixaban reduced the incidence of major bleeding compared with VKAs (Figure S5).

Subgroup analyses based on renal function levels showed that, in terms of major bleeding, compared with VKAs, DOACs reduced the incidence by 21% in patients with CrCl 50–95 ml/min (*P* = 0.02, OR = 0.79, 95% CI 0.64–0.96); by 67% in patients with CrCl 15–29 ml/min (OR 0.33, 95% CI 0.13–0.80). There was no significant difference in patients with CrCl 30–49 ml/min and CrCl < 15 ml/min (*P* = 0.28; *P* = 0.18). There was no heterogeneity between groups for different renal function subgroups (*P* = 0.20, *I*^2^ = 34.8%) (Figure S6).

## Discussion

The current study shows that in patients with AF combined with CKD, DOACs may be able to reduce the incidence of stroke and SE as well as all-cause deaths compared with VKAs in terms of efficacy. In terms of safety, it may reduce the incidence of major bleeding, ICH, fatal bleeding, and minor bleeding, and may not reduce the incidence of GIB and CRNMB. Subgroup analyses showed that [1] in AF patients with comorbid CKD (90 ml/min> Crcl≥15 ml/min), apixaban and rivaroxaban reduced all-cause deaths, apixaban and dabigatran reduced the incidence of stroke or SE, apixaban and edoxaban reduced the incidence of major bleeding, and edoxaban and dabigatran reduced ICH and fatal bleeding, compared to VKAs [2]. In AF patients with comorbid kidney failure, there were no significant differences in the efficacy and safety of rivaroxaban compared with VKAs, except for an advantage in reducing stroke and fatal bleeding, while there were no significant differences in the efficacy and safety of apixaban. Upon excluding direct thrombin inhibitors, specifically dabigatran, the analysis revealed that inhibitors targeting thrombin and factor X can further diminish the occurrence of stroke or systemic embolism, all-cause mortality, massive bleeding, and fatal bleeding in patients with CKD.

In this study, we found that apixaban and dabigatran were superior to VKAs in reducing stroke and SE in patients with CKD and that there was no significant difference between rivaroxaban and edoxaban compared with VKAs. Apixaban and edoxaban reduced the risk of major bleeding significantly compared to VKAs, which is similar to the findings of Feldberg et al. [9], but when compared to VKAs, edoxaban and dabigatran had a significant advantage in reducing ICH and fatal bleeding, and rivaroxaban and apixaban had a significant advantage in reducing all-cause deaths, which was not reported by Feldberg et al. The study conducted by Feldberg et al. included only six RCTs, evaluated only stroke, SE, and hemorrhage, and included aspirin in addition to VKAs in the control group. In contrast, this paper uses 9 RCTs, all of which were conducted in patients with AF, and all of which had VKAs including warfarin as the control drug, making the results more comparable. Comparison with the analysis of Kuno et al. [11] in terms of efficacy. In terms of safety, Kuno et al. concluded that the use of anticoagulants increased the risk of bleeding in dialysis patients, whereas our study showed a similar risk of bleeding. The reason for this may be that Kuno et al. included 16 observational studies, which considered dialysis patients excluded from RCT trials, only 2 studies out of 16 combined AF, and there was a high degree of heterogeneity in the studies. In contrast, 3 RTC trials were included in our study of dialysis patients. RCT trials have strict nadir criteria and the level of evidence for their results is higher than that of observational studies. A study by Chen et al. showed that DOACs was significantly more effective and safer than warfarin in patients with CKD combined with AF [12]. The study by Chen et al. included 6 RCTs and 19 observational studies; there were no RCTs for Kidney failure, and 2 of these studies reported on venous thromboembolism populations non-AF populations. Whereas our article used all RCT studies with a high level of evidence, including 3 RCTs with a CrCl < 15 ml/min. Secondly, Chen et al. analyzed stroke, SE, and VTE together as the same efficacy outcome, which may introduce bias due to the differences in pathomechanisms of SE and VTE. Chen et al. did not conduct further subgroup analysis based on renal function staging and failed to analyze the effectiveness and safety of kidney failure combined with AF patients. The preceding meta-analysis, concentrating on end-stage patients, aligns with our study’s findings [13]. Li et al.‘s investigation encompassed one randomized controlled trial and five observational studies. Despite our inclusion of three randomized controlled trials, it is noteworthy that due to recruitment challenges in some of these trials, the level of evidence in our study did not surpass that of Li et al.‘s. Both investigations illustrated that the use of DOACs, namely rivaroxaban or apixaban, in patients with kidney failure combined with AF yielded comparable efficacy and safety outcomes to those observed with VKAs. A meta-analysis concerning AF patients with kidney failure, comparing DOACs and warfarin, utilized three RCTs [28]; however, one study had missing data [24]. Their outcome measures focused solely on major bleeding, systemic embolism, and cardiovascular death, lacking the depth of analysis found in our article. Additionally, our study included a subgroup analysis of patients with CrCl between 15 ml/min and 90 ml/min, augmenting overall comprehensiveness and persuasiveness. Another meta-analysis on DOACs and warfarin in AF patients with CKD did not specifically explore kidney failure patients with distinct physiological and functional changes [29]. This analysis relied on five pre-2016 RCTs and 14 observational studies. In contrast, a network meta-analysis concluded that DOACs out performed warfarin in preventing thromboembolic events and reducing bleeding risk in AF patients with mild to moderate kidney disease [30]. However, the study acknowledged a limitation in the strength of evidence, precluding a definitive preference for a particular DOACs. Conversely, our study suggests an elevated risk of bleeding without significant benefits from OACs in dialysis patients with AF. In comparison to previous meta-analyses on the efficacy and safety of DOACs and VKAs in AF patients with CKD. This article incorporates nine RCTs with robust evidence levels, encompassing all DOACs. Among these, three RCTs were specifically scrutinized concerning patients with kidney failure, with two employing apixaban and one utilizing rivaroxaban. The use of warfarin in dialysis patients is associated with an increased risk of bleeding, even when maintaining an INR within the normal range, posing challenges to its practical application. Hemodialysis patients typically undergo systemic heparin anticoagulation during their sessions, a variable inconsistently considered in studies examining bleeding complications related to both VKAs and DOACs in this population. As a result, research in this domain has consistently lagged behind. This investigation disclosed no significant disparity in efficacy and safety between VKAs and DOACs for patients with kidney failure and concurrent AF. This finding introduces a novel and more convenient option for anticoagulant therapy in this patient demographic.Our study showed that increased DOACs efficacy and reduced side effects were associated with renal clearance of the drug. Dabigatran has the greatest dependence on renal function due to renal excretion as a prototype [31], apixaban has the lowest renal clearance renal impairment has less effect on its excretion [32], and renal function is moderately affected by rivaroxaban [33]. Compared with warfarin, DOACs has a protective effect on renal function [34]. Our study shows that apixaban may be the best choice when compared to VKAs in the case of an adjusted degree of renal impairment.

Fordyce et al.’s study revealed that the incidence of major bleeding attributed to rivaroxaban at doses of 10 mg daily and 15–20 mg daily in patients with a Crcl of 30–49 ml/min was 6.38% and 4.12%, respectively, with no statistically significant difference observed. In individuals with kidney failure, the incidence of major bleeding increased to 13.04% for those receiving rivaroxaban at a daily dose of 10 mg, and when combined with vitamin K2, the incidence was 9.52%. This underscores the heightened bleeding risk associated with kidney failure. Among patients with a Crcl of 25–30 ml/min receiving apixaban, the incidence of major bleeding for doses of 2.5 mg bid compared to 5 mg bid was 3.42% and 4.39%, respectively, while the incidence of major or clinically relevant non-major bleeding was 4.28% and 7.35%, respectively. The study conducted by A. Mavrakanas et al. concluded that a dosage of apixaban at 2.5 mg bid is the appropriate choice for patients with kidney failure. This suggests a correlation between reduced renal function levels and the necessity for dose reduction.

VKAs is metabolized in the liver by cytochrome P450s and excreted as a metabolite via the kidneys, whereas all DOACs have varying degrees of prototypic drug excretion via the kidneys, and moderate-to-severe renal insufficiency has a significant effect on their pharmacokinetics. Therefore, dose adjustment is required for use in patients with CKD. However VKAs leads to the risk of calcification of the renal arteries, calcification of the aortic valve, and decreased bone calcium [35]. Calcification of small arteries may lead to an increased incidence of ischemic stroke [36]. There is no specific treatment for the defense of calcification due to warfarin anticoagulation, which has a poor prognosis and high morbidity and mortality once it occurs. Warfarin anticoagulation therapy is a risk factor for the development of calcification defense [37]. The US guidelines recommend anticoagulation with warfarin in kidney failure patients with CrCl < 15 ml/min [38]. The nephrology guidelines also recommend warfarin as the drug of choice for anticoagulation in kidney failure patients [39]. However, European guidelines do not recommend DOACs anticoagulation in kidney failure patients with CrCl < 15 ml/min [40]. Previous meta-studies also showed that warfarin for AF patients undergoing dialysis did not show significant benefits or harms [13, 41]. In contrast, our study showed at least similar efficacy and safety when comparing DOACs and warfarin in patients with CrCl < 15 ml/min.

For AF patients with comorbid CKD, since thrombopoietin mRNA can be expressed in the kidneys, decreased renal function can lead to impaired platelet production and an increased risk of bleeding [42]. Inadequate anticoagulation therapy exists for this group of patients, especially dialysis patients, due to the concern that anticoagulant use may lead to an increased incidence of bleeding [43].

This study demonstrates that the use of DOACs improves safety and reduces the incidence of major bleeding, fatal bleeding, and ICH in patients with CKD and concurrent AF, as compared to VKAs therapy. For patients with kidney failure combined with AF, rivaroxaban and apixaban showed at least similar effectiveness and safety when compared to warfarin. This may inform drug selection for oral anticoagulation in patients with CKD combined with AF. Moreover, this article employed nine randomized controlled trials (RCTs), providing a higher level of evidence compared to alternative meta-analyses. Notably, three RCT studies were specifically dedicated to patients with kidney failure, thereby addressing a gap in prior research.

This study also has some limitations: (1) The method of CrCl calculation was not consistent across studies, with 6 studies using the Cockcroft-Gault formula and two using Hemodialysis. (2) Lack of data on the DOACs dose-adjustment regimen, in addition to the level of renal function, the dose of drug use may be affected by factors such as body weight, age, etc., so further studies are needed to investigate the pharmacokinetics of DOACs use in this population. (3) Failure to perform subgroup analysis based on DOACs high and low dose groups.

## Conclusion

Compared with VKAs, DOACs improves the efficacy and safety of anticoagulation in patients with CKD combined with AF. In patients with atrial fibrillation combined with Kidney failure DOACs has at least similar effectiveness and safety when compared with VKAs.

### Supplementary Information

**Supplementary Material 1.**

## Data Availability

All data relevant to the study are included in the article or uploaded as supplementary information.
