# Supplementary material for: Efficacy and safety of direct oral anticoagulants in patients with atrial fibrillation combined with chronic kidney disease: a systematic review and meta-analysis
Source: Thromb J. 2024 Apr 29;22:40. doi: 10.1186/s12959-024-00608-5 (PMC11057070; doi:10.1186/s12959-024-00608-5)
Supplement: Supplementary file 1 — Supplementary Material 1. [file 12959_2024_608_MOESM1_ESM.docx]

**Supplementary material**

**Table S1** Detailed search strategy for each database

| Pubmed (n=1176) |
| --- |
| #1: "renal insufficiency, chronic"[MeSH Terms] OR "renal insufficiency, chronic"[MeSH Terms] OR "renal insufficiency, chronic"[MeSH Terms] OR "kidney diseases"[MeSH Terms] OR "renal insufficiency"[MeSH Terms] OR "kidney failure, chronic"[MeSH Terms] OR "kidney failure, chronic"[MeSH Terms]  #2: "end stage renal disease"[Title/Abstract] OR "chronic renal insufficiency"[Title/Abstract] OR "renal insufficiency chronic"[Title/Abstract] OR "chronic kidney disease"[Title/Abstract] OR "kidney diseases"[Title/Abstract] OR "renal insufficiency"[Title/Abstract] OR "chronic kidney failure"[Title/Abstract]  #3: #1 OR #2  #4: Dabigatran[MESH] OR Rivaroxaban[MESH] OR Factor Xa Inhibitors[MESH]  #5: dabigatran[Title/Abstract] OR Pradaxa[Title/Abstract] OR rivaroxaban[Title/Abstract] OR Xarelto[Title/Abstract] OR apixaban[Title/Abstract] OR Eliquis[Title/Abstract] OR edoxaban[Title/Abstract] OR Savaysa[Title/Abstract] OR non-vitamin K antagonist oral anticoagulant*[Title/Abstract] OR non-vitamin K antagonist*[Title/Abstract] OR DOAC*[Title/Abstract] OR direct oral anticoagulant*[Title/Abstract] OR DOAC*[Title/Abstract] OR novel oral anticoagulant*[Title/Abstract] OR new oral anticoagulant*[Title/Abstract] OR new orally active anticoagulant*[Title/Abstract] OR factor Xa inhibitor*[Title/Abstract] OR factor 10a inhibitor*[Title/Abstract] OR factor IIa inhibitor*[Title/Abstract] OR direct thrombin inhibitor*[Title/Abstract]  #6: #4 OR #5  #7: #3 AND #6 |
| Web of science (n=1821) |
| #1: TS= “dabigatran” OR TS= “Pradaxa” OR TS= “rivaroxaban” OR TS= “Xarelto” OR TS= “apixaban” OR TS= “Eliquis” OR TS= “edoxaban” OR TS= “Savaysa” OR TS= “non-vitamin K antagonist oral anticoagulant*” OR TS= “non-vitamin K antagonist*” OR TS= “DOAC*” OR TS= “direct oral anticoagulant*” OR TS= “DOAC*” OR TS= “novel oral anticoagulant*” OR TS= “new oral anticoagulant*” OR TS= “new orally active anticoagulant*” OR TS= “factor Xa inhibitor*” OR TS= “factor 10a inhibitor*” OR TS= “factor IIa inhibitor*” OR TS= “direct thrombin inhibitor*”  #2: TS= "end stage renal disease" OR TS= "kidney failure, chronic" OR TS= "chronic renal insufficiency" OR TS= "renal insufficiency chronic" OR TS= "chronic kidney disease" OR TS= "kidney diseases"[Title/Abstract] OR TS= "renal insufficiency" OR TS= "chronic kidney failure" OR TS= "renal insufficiency, chronic" OR TS= "renal insufficiency, chronic" OR TS= "renal insufficiency, chronic" OR TS= "kidney diseases" OR TS= "renal insufficiency" OR TS= "kidney failure, chronic"  #3: #1 AND #2 |


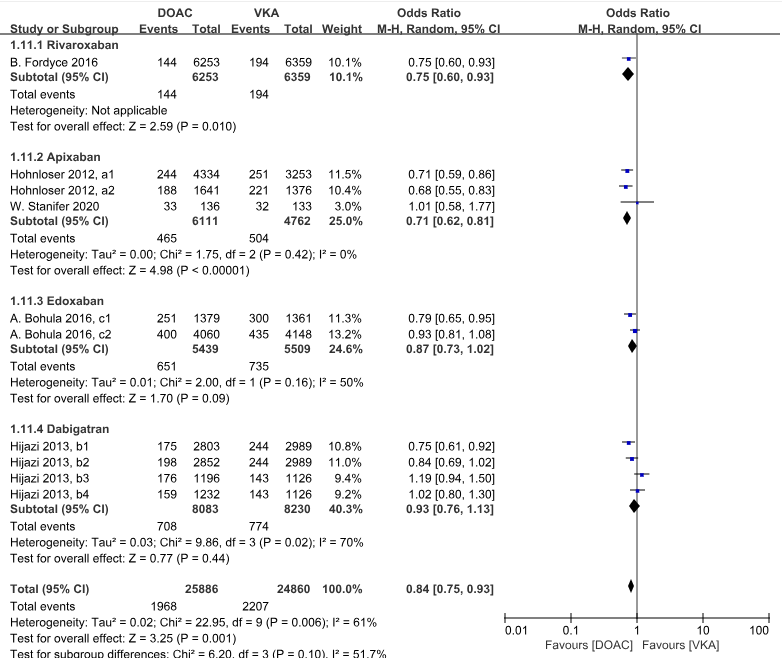


**Figure S1** Forest plot of all-cause death subgroup analysis according to DOAC in patients with CKD combined with AF. a1: CrCl 51-80 ml/min, apixaban 5 mg twice daily or 2.5 mg twice daily. a2: CrCl≤50 ml/min, apixaban 5 mg twice daily or 2.5 mg twice daily; b1: CrCl 50-80 ml/min, dabigatran 110 mg twice daily; b2: CrCl 50-80 ml/min, dabigatran 150 mg twice daily; b3: CrCl 30-49 ml/min, dabigatran 110 mg twice daily; b4: CrCl 30-49 ml/min, dabigatran 150 mg twice daily;


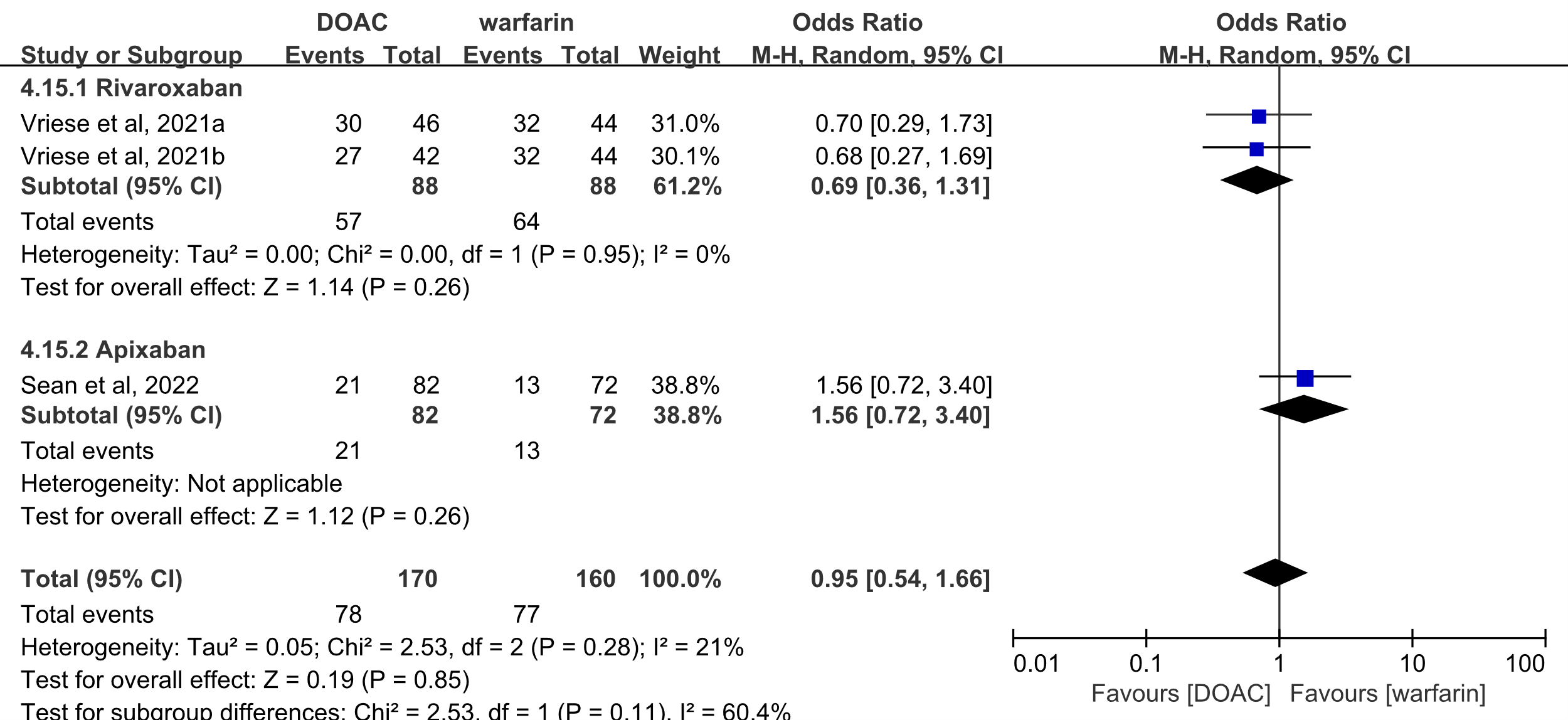


**Figure S2** Forest plot of all-cause death subgroup analysis according to DOAC in patients with kidney failure combined with AF. d1: CrCl＜15 ml/min, rivaroxaban 10mg daily; d2: CrCl＜15 ml/min, rivaroxaban and vitamin K2.


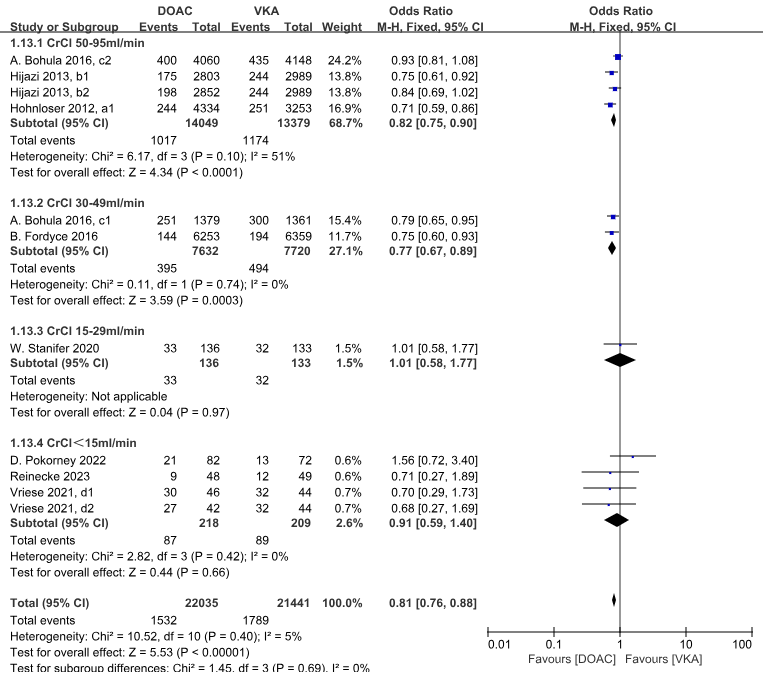


**Figure S3** Forest plot of all-cause deaths subgroup analysis according to renal function staging. a1: CrCl 51-80 ml/min, apixaban 5 mg twice daily or 2.5 mg twice daily; b1: CrCl 50-80 ml/min, dabigatran 110 mg twice daily; b2: CrCl 50-80 ml/min, dabigatran 150 mg twice daily; c1: CrCl 30-50 ml/min, edoxaban 30 mg daily; c2: CrCl 50-95 ml/min, edoxaban 60 mg daily; d1: CrCl＜15 ml/min, rivaroxaban 10mg daily; d2: CrCl＜15 ml/min, rivaroxaban and vitamin K2.


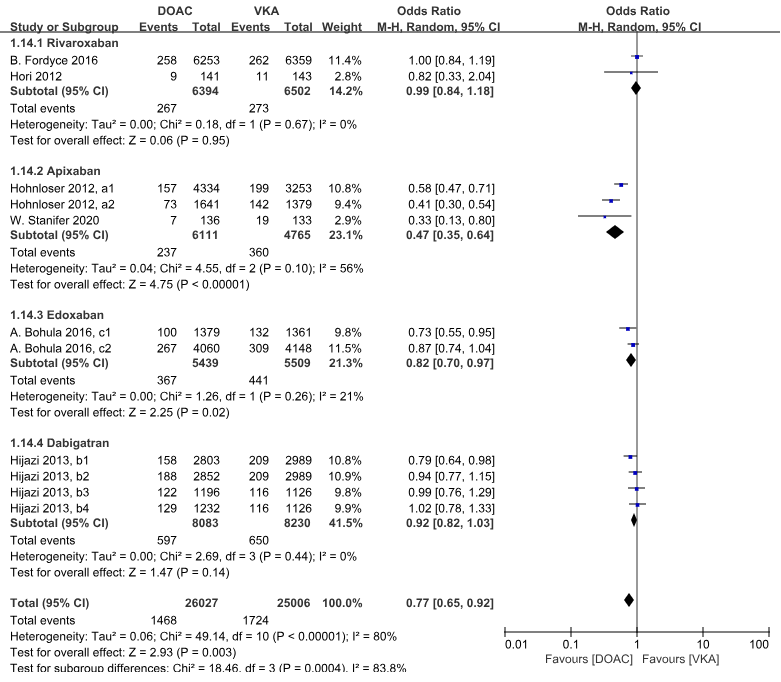


**Figure S4** Forest plot of CKD combined with AF major bleeding according to DOAC subgroup analysis. a1: CrCl 51-80 ml/min, apixaban 5 mg twice daily or 2.5 mg twice daily; a2: CrCl≤50 ml/min, apixaban 5 mg twice daily or 2.5 mg twice daily; b1: CrCl 50-80 ml/min, dabigatran 110 mg twice daily; b2: CrCl 50-80 ml/min, dabigatran 150 mg twice daily; b3: CrCl 30-49 ml/min, dabigatran 110 mg twice daily; b4: CrCl 30-49 ml/min, dabigatran 150 mg twice daily; c1: CrCl 30-50 ml/min, edoxaban 30 mg daily; c2: CrCl 50-95 ml/min, edoxaban 60 mg daily.


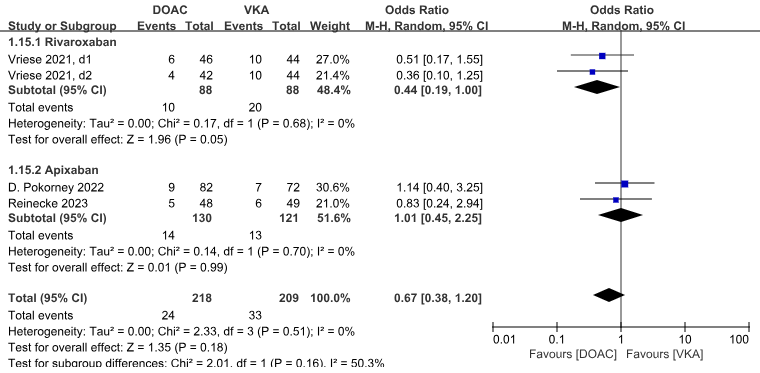


**Figure S5** Forest plot of ESRD combined with AF major bleeding according to DOAC subgroup analysis. d1: CrCl＜15 ml/min, rivaroxaban 10mg daily; d2: CrCl＜15 ml/min, rivaroxaban and vitamin K2.


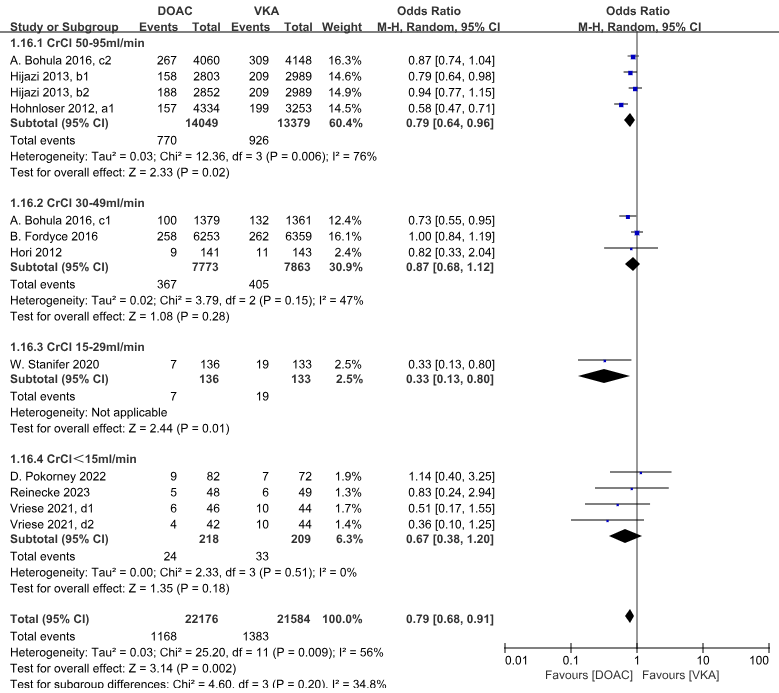


**Figure S6** Forest plot for subgroup analysis of major bleeding according to renal function staging. a1: CrCl 51-80 ml/min, apixaban 5 mg twice daily or 2.5 mg twice daily; b1: CrCl 50-80 ml/min, dabigatran 110 mg twice daily; b2: CrCl 50-80 ml/min, dabigatran 150 mg twice daily; c1: CrCl 30-50 ml/min, edoxaban 30 mg daily; c2: CrCl 50-95 ml/min, edoxaban 60 mg daily; d1: CrCl＜15 ml/min, rivaroxaban 10mg daily; d2: CrCl＜15 ml/min, rivaroxaban and vitamin K2.
